# Environmental Synthesis of Few Layers Graphene Sheets Using Ultrasonic Exfoliation with Enhanced Electrical and Thermal Properties

**DOI:** 10.1371/journal.pone.0152699

**Published:** 2016-04-11

**Authors:** Monir Noroozi, Azmi Zakaria, Shahidan Radiman, Zaidan Abdul Wahab

**Affiliations:** 1 Department of Physics, Faculty of Science, Universiti Putra Malaysia, Serdang, Selangor, Malaysia; 2 School of Applied Physics, Faculty of Science and Technology, Universiti Kebangsaan Malaysia, Bangi, Selangor, Malaysia; Tsinghua University, CHINA

## Abstract

In this paper, we report how few layers graphene that can be produced in large quantity with low defect ratio from exfoliation of graphite by using a high intensity probe sonication in water containing liquid hand soap and PVP. It was founded that the graphene powder obtained by this simple exfoliation method after the heat treatment had an excellent exfoliation into a single or layered graphene sheets. The UV-visible spectroscopy, FESEM, TEM, X-ray powder diffraction and Raman spectroscopy was used to analyse the graphene product. The thermal diffusivity of the samples was analysed using a highly accurate thermal-wave cavity photothermal technique. The data obtained showed excellent enhancement in the thermal diffusivity of the graphene dispersion. This well-dispersed graphene was then used to fabricate an electrically conductive polymer-graphene film composite. The results demonstrated that this low cost and environmental friendly technique allowed to the production of high quality layered graphene sheets, improved the thermal and electrical properties. This may find use in the wide range of applications based on graphene.

## Introduction

Recently, graphene as a single atomic layer of carbon has attracted much attention due to its unique nanostructure and novel thermal, electric and mechanical properties as well as its potential applications in physics, materials science, chemistry and biology [1]. Graphene has an electrical conductivity greater than any other known material [2], and its thermal conductivity is higher than that of diamond [3]. However, the properties of graphene have been challenged by issues in the production of graphene sheets by synthesis whether single or few-layered, as it is usually difficult to achieve on a large scale production. In 2010 Novoselov et al were awarded the Nobel Prize for their experiment regarding the preparation of monolayer graphene [4]. Bottom-up and up-bottom methods are two major approaches for the preparation of graphene materials. In bottom-up methods, high quality of graphene can be obtained however in a low quantity, such as chemical vapor deposition (CVD) [5] and annealing SiC substrates [6]. Later method implies that the graphene can be produced in the form of low or free defect sheets, based on exfoliation methods [7] mainly including mechanical, electrochemical and chemical exfoliation of graphite oxide [8] and liquid phase exfoliation of graphite (GT) towards graphene [9]. The most common liquid phase exfoliation technique is the chemical reduction of the exfoliated graphite oxide by sonication [10] or heat treatment is the most promising technique to produce a few layer reduced nanographene [11,12]. However, the oxidization and exfoliation process a produced only very small graphene sheets with a large number of defects [13]. Additionally, concentrated acids are needed in oxidisation and the harsh reduction processes that are used to reduce graphite oxide are more expensive, with high environmental risks [14]. One possible solution to address this problem was the usage of liquid exfoliation method (without oxidation). The simplest effect of exfoliation is to dramatically decrease the Van der Waals forces between the layers and produce single or few layer of graphene, this can in turn, radically enhance the electronic, mechanical, thermal and chemical properties for coatings, composites, and other graphene material applications [15]. Although technically this method is similar to the chemical reduction of the exfoliated graphite oxide, it is unique because of the minimal defect concentration by direct exfoliation of graphite in solution and low oxygen functional groups. Several studies of liquid exfoliation have been reported to produce graphene dispersions in common chemical solvents in the presence of surfactants by ultrasonication, due to its relative simplicity, scalability, and the quality of graphene produced [16]. However, some issues such as relative stability, long sonication periods, expensive solvents and the excessive use of surfactants are limiting factors in the production of the graphene layers [17]. Industrial solvents and surfactants have some disadvantages, e.g. NMP and DMF have toxic effects, and most of them have high boiling points, hence making it difficult to remove the solvents after exfoliation [18,19]. Therefore, the key challenge in producing graphene sheets was the exfoliation of graphite in a suitable solvents or surfactants that could disperse highly stable graphene sheets in large quantities. Unfortunately, dispersion of graphehe in water as its solvent is one of the critical steps involved in the exfoliation of graphite, due to the hydrophobic nature of graphite. The use of polyvinylpyrrolidone (PVP) as a polymer stabilizer, that is bio–compatible, inexpensive and soluble in water, has shown promise for stabilizing the dispersion of graphene in the solvents that normally cannot be dispersed, such as chloroform, isopropanol, and hexa fluoroisopropanol (HFIP) [20–22]. In this work, we selected locally produced liquid hand soap as a green alternative surfactant rather than an industrial surfactant for exfoliating graphite in water in the presence of PVP, by using a high intensity probe-sonication. Hand soap, composed of a mixture of ash, water and oil, mixed to get a soap-like substance, which offered strong solubility in water, low viscosity and high solution stability [23,24]. Although, graphite has been exfoliated in a household dish-washing fluid using a high shear rotor/stator laboratory [25] and a kitchen blender [26]; yet, ultrasonic exfoliation is an advantageous method to use as an alternative for the fabrication of few layer graphene, with a high quality [27,28].

We suggest that the molecules in hand soap fluid may have similar interactions with graphite flakes to produce single or few layers graphene sheets. For testing this, hand soap—PVP (SP), as the solvent and dispersant and stabilizer, was selected to prepare a stable dispersion of the individual graphene sheets in water by using a high power sonicator. Benefits of hand soap include its low cost, established safety, non-pollutive properties and hence, a low environmental risks. In this Article, the probe ultrasonication technique produces the stable dispersions of a few layered graphene in SP solution with low defects, hence resulting in a high electrical conductivity. After wash, dry and thermal treatment, we obtained pure graphene powder, with high quality and with a low defect ratio. Kuang et al and other research groups [29–31] have investigated the thermal properties of graphene and graphene composite. Their results indicated that while single layer graphene has high thermal conductivity, with increasing number of layers the thermal conductivity of multilayer graphene can be significantly reduced to eventually approach the value of bulk graphite. Huan et al [32] measured the thermal diffusivity of a two layer graphene by using the transient electro thermal technique. They found that the thermal diffusivity of two layer graphene, 1.16–2.22 × 10^−4^ m^2^ s ^−1^, was about one order of magnitude lower than single layer graphene. Gupta et al [33] found that the nanofluid produced by liquid phase exfoliation technique, contains a few layers of graphene which has a higher thermal conductivity than that of the base fluid. This enhancement can improve with the increase in concentration; however there are no reports on thermal diffusivity of the exfoliated graphene nanofluids. Although thermal properties of graphene dispersion are intensely researched, studies on thermal conductivity are more common in literature [34–36].To our knowledge, this is the first time that the thermal diffusivity of graphene dispersion has been investigated. The thermal diffusivity of the resulting graphene dispersion was measured using the Thermal-Wave Cavity (TWC) methodology, a simple approach of Photothermal techniques, to measure the thermal diffusivity of liquid samples with high accuracy [37–39]. The resulting graphene dispersion in SP was used to fabricate a SP polymer-graphene film, for testing its electrical conductivity. It was found that graphene in the SP solution has excellent thermal diffusivity and it could be used to produce graphene based films for conductive transparent film in electrical devices. The overall results of the graphene dispersions described here indicate that can easily produce high-quality graphene with low cost. This simple method could be used for the production of high-quality graphene, and this graphene preparation could be used in the wide range of applications, such as in thermal devices, electronics, printable electrochemical sensors, and energy storage devices.

## Experimental Details

### Materials and methods

The pure graphite powders (+100 mesh, i.e. >150 μm particles) and Polyvinyl pyrrolidone (PVP, Mw: 30, 000) were purchased from Sigma- Aldrich company.

Solvent: Liquid hand soap (Dettol Original Liquid Hand Wash, Reckitt & Colman Ltd., U.K) was dissolved in deionized (DI) water to create a solution of hand soap-DI water, 0.5 wt%. For exfoliation and dispersion of graphite in the solution, a probe ultrasonic homogenizer (UH) (provided in continuous or pulse mode, 0–300 watts, Model 3000, Biologics, Inc., Virginia, USA) was used with high intensity. The sonication probe tip was provided in continuous mode for 60% amplitude modulation.

### Preparation of the exfoliated graphene

**Fig 1:**
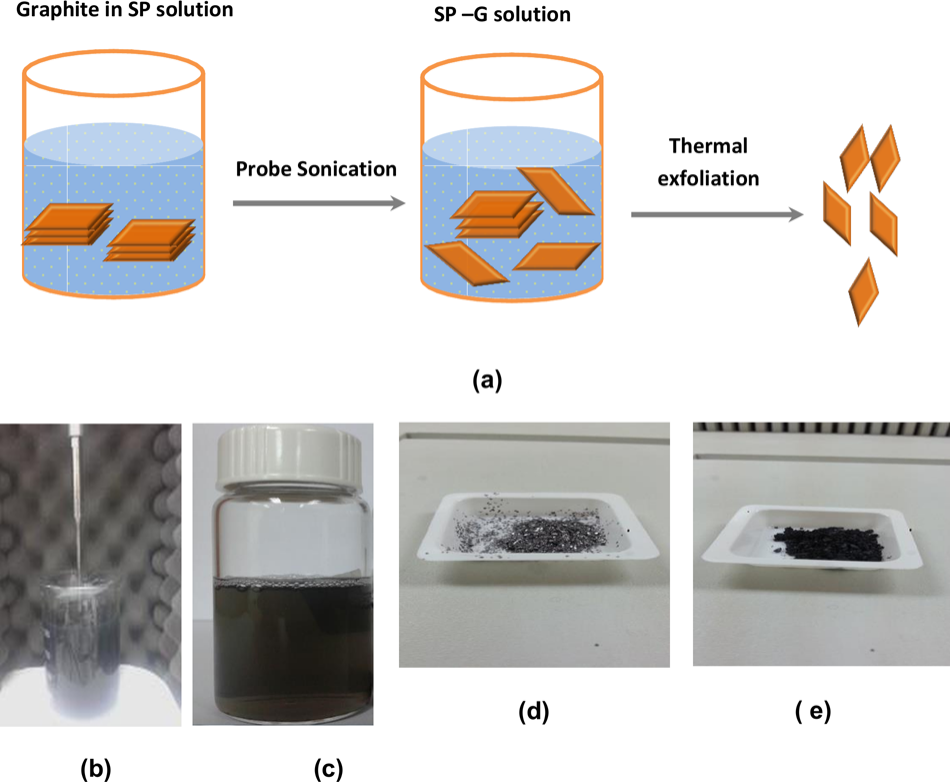
(a) Schematic illustration of the exfoliated graphite in SP solution and the synthesis of graphene powder; (b) The mixture of graphite in SP solution was sonicated using a probe ultrasonic homogenizer to produce stable SP-G solution; (c) The stable dark SP-G dispersions that remained of high quality even after several months; (d) The shiny metallic grey colour of the starting graphite powder; (e) The resulting black G powder after heat treatment.

The mixture, SP, containing 1g PVP with 400 ml of hand soap-DI water (0.5 wt%) was stirred for 1 h, then, 1 g of pure graphite (GT) powder was added to the SP solution to form graphite solution. The resulting solution was tip sonicated for 3 hrs, and then was left to stand for 24 hrs to separate the large graphite aggregates to the bottom. The homogenous dark dispersion remained suspended for at least five months after preparation without any significant precipitation (as shown in Fig 1B). This solution was denoted as SP-G and was collected for further uses and characterization. To totally remove SP and any non-exfoliated graphite, the sample was washed with DI water and was further isolated by centrifugation at 4,000 rpm for several hours. Next, the SP-G gel was obtained after increasing the concentration of graphene particles and the resultant sample was dried in a vacuum oven at 80°C overnight. For further treatment, the final product was subjected to thermal annealing at 1000°C for 5 min to remove any residual SP from the graphene/graphite and the resulting black powder was denoted as G, containing pure graphene and was kept for further characterization.

### Fabrication of graphene composite film

The liquid exfoliation of graphene can easily be formed into films for a wide range of electronic applications. In order to study the electrical conductivity effects, the SP-G solution was used to prepare the graphene polymer composites films, by using a spin coating method. A small volume of SP-G (≈100 μL) was dropped on the substrate during the spin coating process at 1000 rpm for 30 s, and finally, the film was subjected to heat treatment at 100°C under vacuum for 48 hrs to remove the solvent remnants and produce the multi-layered graphene. The quartz substrates were washed by distilled water and acetone and then placed in an ultrasonic cleaner for 30 min for the cleaning process.

## Characterization

The optical absorption of the diluted solution before and after ultrasonic treatment was analysed using an UV−vis spectrophotometer (Perkin Elmer model Lambda 900). The powder morphology of the pure GT, the resulting graphitic powder and films were determined by field-emission scanning electron microscopy (FE-SEM; SUPRA 55VP, Carl Zeiss, Jena, Germany) and transmission electron microscopy (TEM; Philips CM12, Amsterdam, The Netherlands; operated at 100 kV) and X-ray diffraction (XRD) (Bruker D8 Advance, Karlsruhe, Germany) equipped with a Cu Kα (40 kV, 40 mA) radiation source. Raman spectroscopy was performed (532 nm laser, WiTec brand, model Alpha 300J, Germany) which was used to analyze edge defects and estimate layer thickness. X-ray photoelectron spectroscopy (XPS) measurement was carried out using a Multifunctional XPS (Shimadzu Axis Ultra DLD). The electrical resistance of graphene composite film was characterized by four point probe measurement using a Keithley 2401 source meter.

### Thermal diffusivity measurement

The experimental TWC technique for measuring the liquid thermal diffusivity was utilised as reported previously [39]. In this method, the modulated beam light of a diode laser (532 nm, 200 mW, (MGL(10)) from a chopper (SR540) was absorbed and converted to thermal waves (TW) by a thin metallic black painted foil (50μm thickness). The resulting TW was transferred into the liquid sample and the thermal content was detected using a very sensitive pyroelectric (PE) detector, polyvinylidene diflouride PVDF film (52 micron, MSI DT1-028K/L). The generated PE signal was analyzed by using a lock-in amplifier (SR 530). The PE signal can be obtained by varying the laser modulation frequency in the range 5–40 Hz and was carried out at room temperature (~20°C). The PE signal *V* was determined by the thickness and thermal diffusivity of sample, *V*(*f*) = *V*_0_e^−*σL*^ where *V (f)* is the complex PE signal, σ is the complex TW diffusion coefficient and *L* is the liquid sample thickness. From a relation *σ* = (1 + *i*)/*μ*, the thermal diffusion length of sample is *μ* = (*α*/*π f*)^1/2^, where *f* is the modulation frequency, and *α* is the thermal diffusivity of sample. The amplitude and phase of the signal are respectively, [38]

$$|V(f)|=V_{0}\left(f\right)\;\times e^{-L\;/\mu }$$ (1)

$$\varphi (f)=\varphi_{0}\left(f\right)-L/\mu$$ (2)

From the graph of the ln(amplitude) and phase as a function of the square root of the frequency, the thermal diffusivity of liquid sample can be calculated from the slopes of *S* = *L*(*π*/*α*)^1/2^.

## Results

### The exfoliated graphene dispersion

Fig 1(A) shows the schematic illustration for the exfoliation of graphite in SP solution and synthesis of graphene powder. The mixture of graphite in SP solution was sonicated using a probe ultrasonic homogenizer (Fig 1B) to produce a stable and well dispersed solution of graphene. Fig 1C shows the stable dark SP-G dispersions that remained of high quality even after several months. The resultant solutions indicated that the graphene sheets were well dispersed in solution. After centrifugation, washing and drying, the SP-G was subjected to heat treatment to remove any residual SP to obtain black pure graphene powder (denoted as G). Fig 1D and 1E show the shiny metallic grey colour of the starting graphite and the resultant black graphene powder after heat treatment.

**Fig 2:**
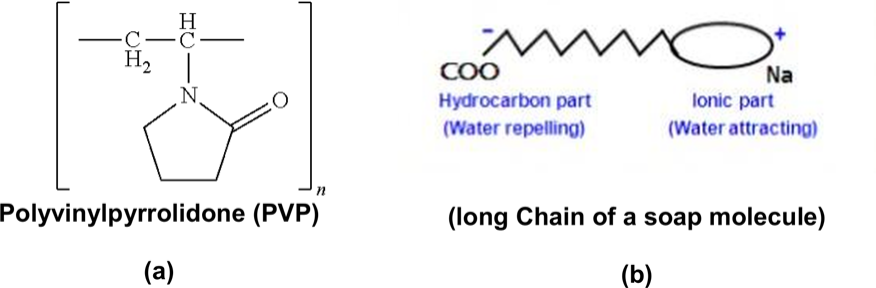
**(a) Chemical structures of PVP and (b) long chain of a soap molecule has two parts, polar and non-polar structures,** used as stabilizer and surfactants in the process of liquid-phase exfoliation of graphite towards graphene.

The extent of exfoliation for graphite could be increased with increased ultrasonication time, due to the high energy activation of ultrasonication [40]. This activation is needed to overcome interlayer Van der Waals forces, therefore, the selection of a suitable solvent and diffusion of a suitable surfactant and stabilizer are needed for decreasing the depth of the Van der Waals interactions and increasing the stability of the exfoliated graphene during the exfoliation process [41]. The long chain of a soap molecule has two parts, polar and non-polar, i.e a long hydrophobic "tail" (water-fearing) and a hydrophilic "head" (water-loving) that is anionic, as illustrated in Fig 2. Soap, as a surfactant, is able to increase the ability of water to dissolve graphite. A small amount of hand soap in water could indeed be an effective method to reduce the thickness of graphitic flakes and increase the exfoliation yields of graphite which is dependent on a high energy, process and the length of the molecular chain of the soap molecule [41]. The chemical structure of PVP is shown in Fig 2. PVP as a dispersant and stabilizer was selected, due to fact that it is highly stable in the graphene dispersion during the exfoliation [42,43].

**Fig 3:**
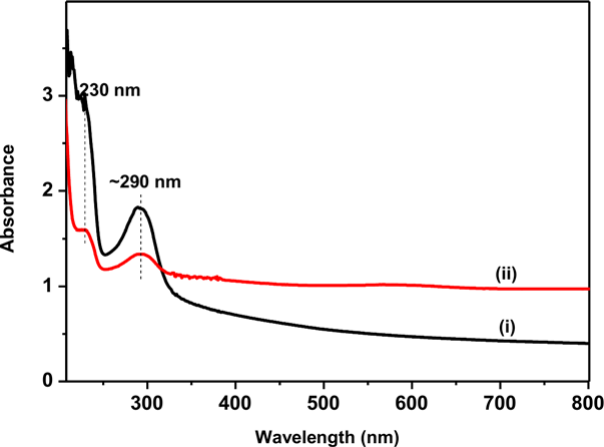
UV-vis absorption spectrum of the SP-G solution before (i) and after (ii) ultrasonic treatment.

For investigating the graphene exfoliated by sonication treatment, the optical absorption of the SP-G solution before and after sonication of 3 hrs was analysed, Fig 3 shows the UV-vis absorption of the SP-G solution (i) before and (ii) after ultrasonic treatment. Interestingly, it was found that although the spectra of both solutions had characteristic peaks in the UV range, however, the maximum absorption at 230 nm was reduced after ultrasonic treatment, due to the reduction of the C = O groups [44]. Additionally, after sonication, no significant peak was observed at 320 nm (characteristic of graphene oxide), in the dispersion solutions, suggesting that ultrasonic treatment prevent significant generation of graphene oxide [44]. The absorbance peak centred at ~290 nm was attributed to the excitation of the π–plasmon absorption of the C = C bonds of the graphitic structure [45], similar to that in carbon nanotubes, which indicated the successful incorporation of the graphene into before (i) and after (ii) ultrasonic treatment. However, the peak obtained in the electronic spectra of the SP-G prepared with ultrasonication, (ii), was much broader, with a full-width-half-maximum (FWHM), at 35 nm (compared to 27nm in (i)). It was observed that the peak decreased with a decrease in the thickness of graphene sheets, and the low intensity of the adsorption peak in sample (ii) demonstrated that ultrasonic treatment could increase the exfoliation of graphene solution to the reduced flake size of graphite. As previously observed for the exfoliation of graphene in a solvent, with an increasing sonication time, thickness of the sheets is reduced; however, at longer sonication time, it can be reduced to the thickness of the sheets and also affect the quality of graphene for using in several applications[46].

**Fig 4:**
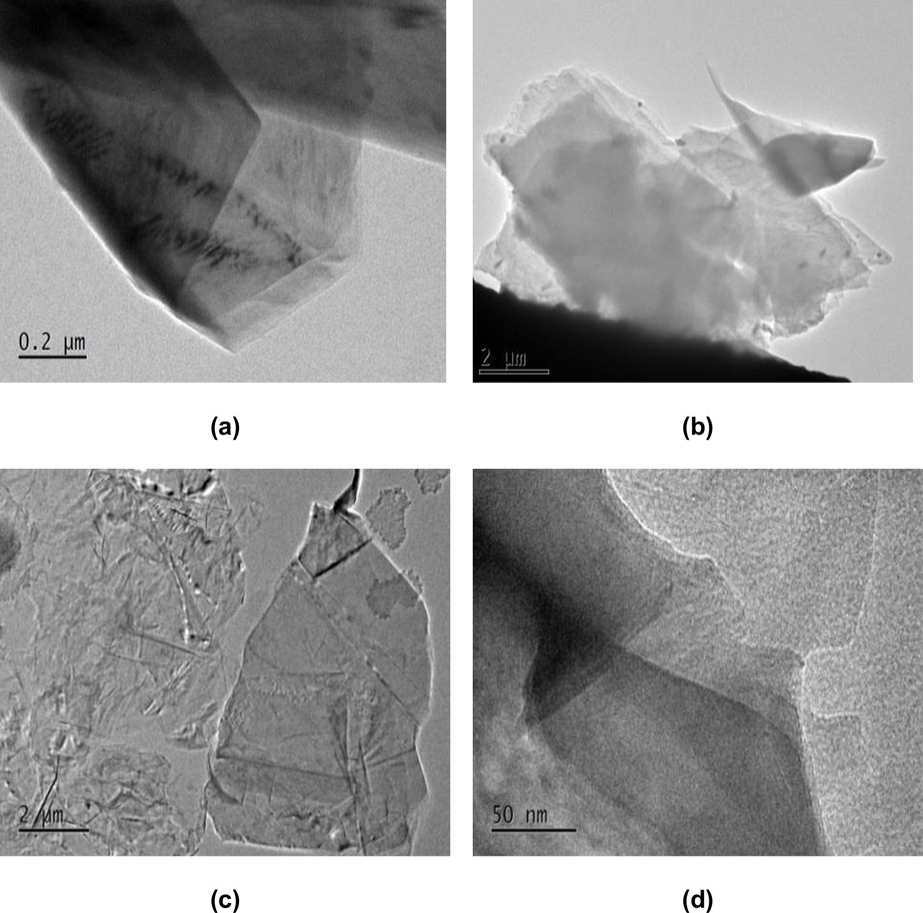
TEM analysis of the SP-G solution obtained by sonication. **(a,b)** multilayer (low quality), **(c)** monolayer and very large sheet (high quality), **(d)** high resolution TEM.

TEM was employed to show the quality and morphology of the SP-G solution prepared with the ultrasonic method. Fig 4 shows that this suspension contained well exfoliated graphene sheets, including multilayers (Fig 4A and 4B) and some monolayers, with very large sheets (Fig 4C and 4D). Fig 4A and 4B show clearly partially exfoliated graphite flakes with some aggregation and also multilayer graphene sheets. The monolayer graphene sheet with thin flat graphene flake and large dimensions is shown in Fig 4C. From the high resolution TEM image in Fig 4D, there are evidence that some of the flakes observed in the survey were clearly monolayers of graphene. The results indicated that the resultant graphene sheets are dispersion of single and few layer graphene sheets, which were well exfoliated without significant structural defects, so this process could be scaled up successfully.

### Structure of the exfoliated graphene

**Fig 5:**
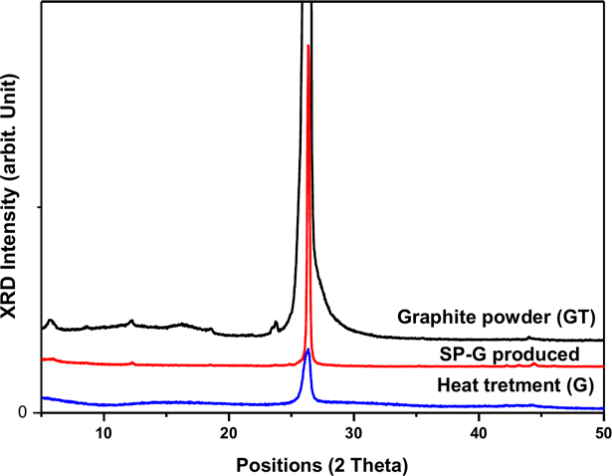
XRD curves of the pure GT, the prepared samples without (SP-G) and with (G)heat treatment.

The XRD patterns of pure GT, and the prepared samples without (SP-G) and with (G) heat treatment are displayed in Fig 5. The pure GT has a strong intense peak at a 2θ of about 26.5° corresponding to (002), which is higher than SP-G and G. On conversion to SP-G and G, there is a decrease in the intensity and the peak becomes broad in comparison with natural graphite, which means that the inter planar carbon bonds get broken and the crystalline size of graphite is reduced [46]. However, this decrement was more obvious in the sample after heat treatment, these results show that the graphene sheets in the exfoliated graphene after heat treatment, G, has more ordered structures than that before heat treatment, SP-G.

**Fig 6:**
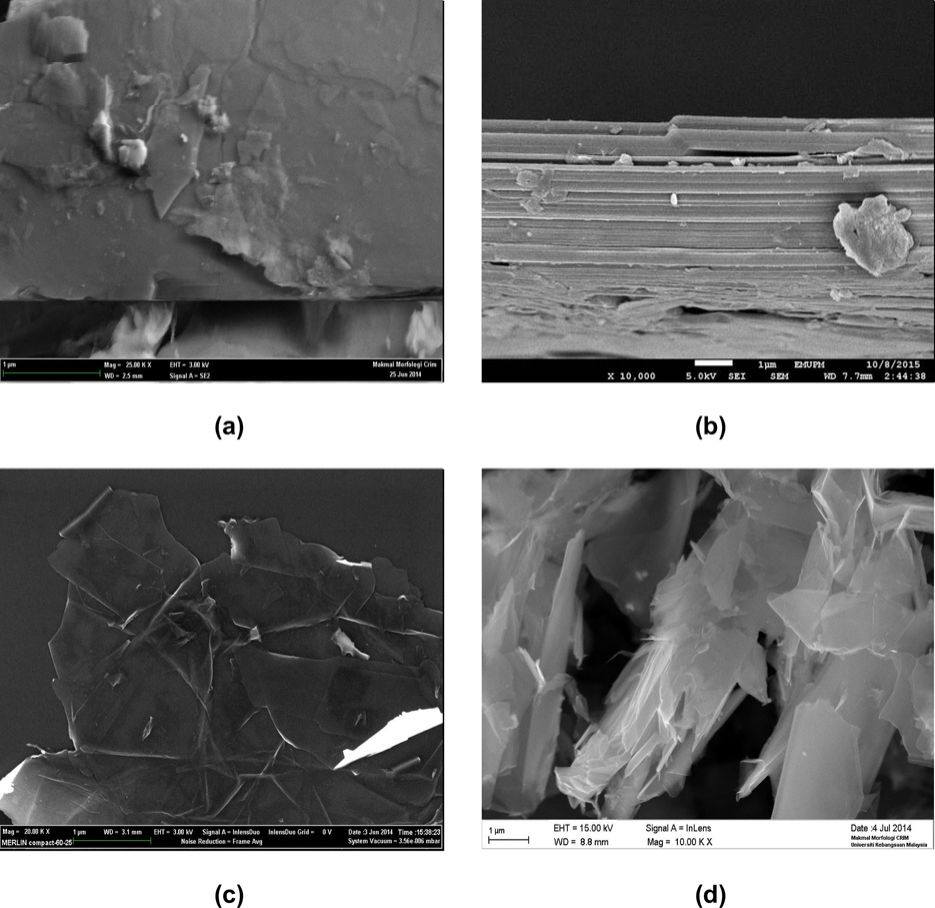
SEM image of (a, b) pure GT powder (c, d) the prepared G powder after heat treatment.

Fig 6 shows the FESEM images of the pure GT and the resulting G powder produced after heat treatment. Fig 6A and 6B show the GT have rigid sheets with a smooth surface and thick layers ranging from a few hundred nanometers to several micrometers. The images of the G sample after heat treatment, (Fig 6C and 6D) implied that the interlayer spacing was increased and the graphene nanosheets were thin enough to become semi-transparent with only a single or few layers, and the nanosheets layers had sharp corners with well-defined edges. In Fig 6C the surface of sheet was very smooth and free of defects. This result was similar to data for the graphene powder obtained through intercalation and thermal shock methods [47].

**Fig 7:**
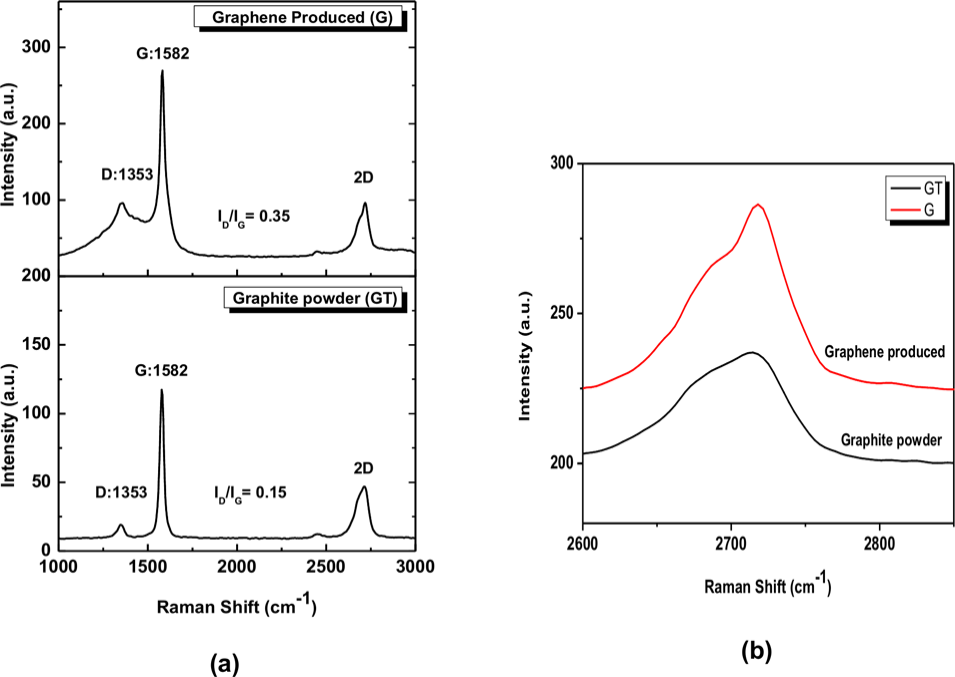
Raman spectra of pure GT and the exfoliated G powder after heat treatment. (a) Raman spectra of (top) pure GT powder and (bottom) the G powder produced after heat treatment (G); b) The magnified 2D bands of (top) G and (bottom) GT.

Raman Spectroscopy is a powerful tool to determine the number of layers present along with the quality of the graphene present (relative defects in graphene) with a faster throughput than other techniques such as AFM [19]. Fig 7A shows the Raman spectrum of (top) the prepared G powder, with (bottom) the spectrum of the pure GT powder, that is shown for comparison, (b). The magnified 2D band intensity of the two Raman spectra is compared. As seen in Fig 7A, the Raman spectra of both two samples, GT and G, showed two prominent peaks at ~1350 and ~1580 cm^-1^, which corresponded to the D band and G band, respectively. The D band at 1350 cm^-1^ corresponds the breathing mode of sp^2^ carbon atoms, and it is absent without the presence of defects and edges in the graphitic structure [12]. The pure GT powder shows a small number of defects (GT_ID/IG_ = 0.15), while as expected a small ratio (G_ID/IG_ = 0.35) is found for the exfoliated graphene sheets. We propose that the ratio of the defects G_ID/IG_ to the defect GT_ID/IG_ (i.e. M = G_ID/IG_ /GT_ID/IG_) can be used to quantitatively estimate the graphene sheets. We suggest using the ratio of the intensity of the D: G band ratio of graphene sheets, divided by the intensity of the D: G band ratio of the graphite source. This relationship (the value of M) allows the measurement of the mean defects of the graphene sheets from the graphite used to produce the graphene. The edge defects factor of the exfoliated graphene in Fig 7B was around M = 2.3, the data from Paton [25] was around 2.2 and from Ou et al. [48] were in the range of 2.3–6.4. The results indicate that small quantities of defects were introduced during the exfoliating process, which has a significant effect on the quality of the graphene sheets as well as the properties of graphene material. This low defect ratio is indicative that the sonication process induced some defects into the graphene sheets; however there was only a low concentration of edge defects in the graphene sheets and it was well exfoliated without significant structural defects. It has long been known that the shape of the 2D Raman band (~2700 cm^-1^) is an effective means to determine the number of layers in the graphene sheets. As seen in Fig 7B, compared to graphite, the 2D band of the exfoliated graphene became symmetrical and sharp, suggesting that the resultant graphene proved the successful exfoliation of graphene with a few layers (thinner than five layers) [48]. The shape of the 2D band in Fig 7B is similar in shape of the spectra published by Paton et al [25], and Yoon et al [49] for 4–5 layer graphene. This was in agreement with the results that were seen by TEM and SEM imaging, that the graphene product could also have some amount of single layer graphene, as presented in Figs 4C and 6C.

**Fig 8:**
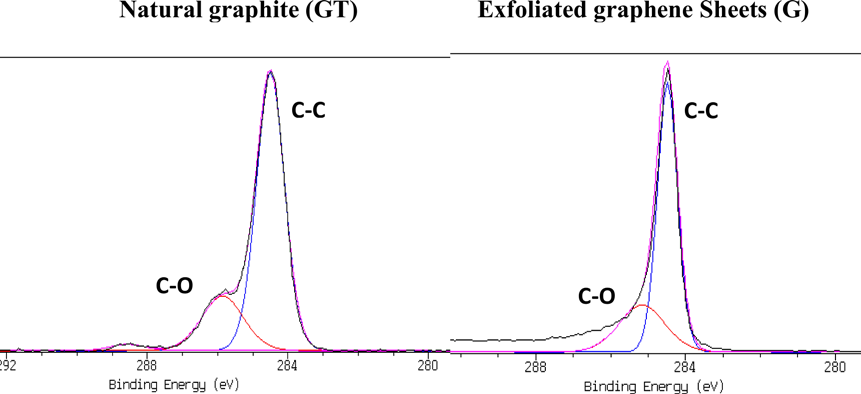
high resolution spectra for C1s peak of the pure GT powder (left) and the obtained G powder (right).

To further evaluate the quality and also the oxygen containing groups in the sheet, XPS analysis was performed for the pure GT and the G powder obtained by sonication after heat treatment. The high resolution of the sharp and large carbon 1s (C1s) peak of GT and G represented the graphitic carbon sp^2^ (C-C) and consisted of peaks corresponding to carbon bound (C–C) at ~284.5 eV and (C-O) at around 286 eV, respectively [50,51] (Fig 8). Therefore, it can be concluded that no obvious changes occurred on the chemical structure of the pure GT powder and the G sample obtained after heat treatment. However, the XPS analysis of the G, exfoliated graphene sample, shows a negligible reduction in the peak intensity of epoxide bonds (C–O bonds). This reduction could be the reason of the sonication process and the heat treatment in the obtained G sample[17,18,46]. This result can be explained by the fact that the graphite in the final material was exfoliated into the graphene sheets. This observation was also in good agreement with the Raman and UV-vis spectroscopic data.

### Electrical conductivity of graphene composites film

**Fig 9:**
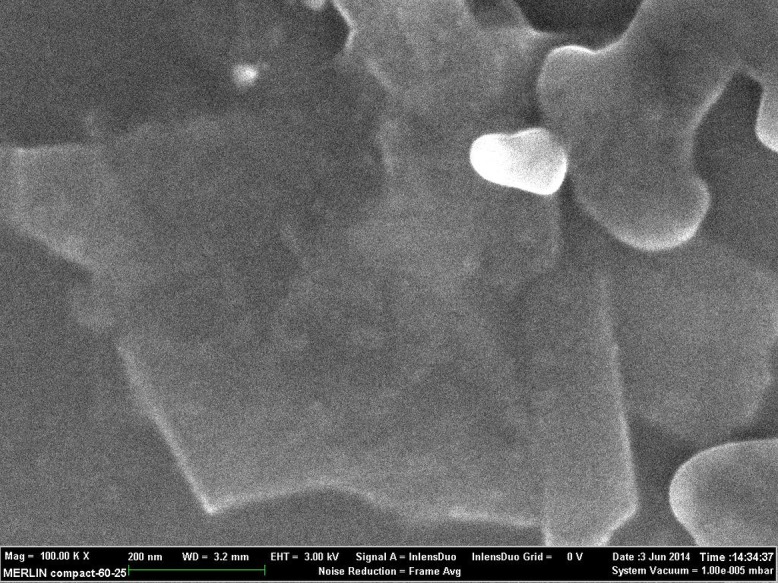
FESEM images of SP-G composite film, some of the graphene sheets had a large surface with the size of the sheets at a few microns.

This section presents the morphology and graphene surfaces of the SP-G film as a graphene/polymer composite and also its electrical conductivity. Fig 9 shows the SEM images of the SP-G composite films deposited on substrate. The film was prepared by a vacuum spin coater onto the quartz substrate. As presented in Fig 9, it was found that the distance or space between the graphene sheets was on a micrometer scale and some of the graphene sheets had a large surface with one or few -layer graphene sheets, where the size of sheets was about a few microns with no overlapping observed. As revealed by the result, the successful exfoliation of graphite was found to produce homogeneous graphene with few-layer nanosheets within the SP mixture.

For testing the electrical conductivity, the sheet resistance of the SP film with and without graphene loading are shown in Table 1. The sheet resistance of pure SP was high (> MΩ per square), while, the sheet resistance (Rs) of the film with graphene was reduced dramatically to 1.5 kΩ per square, by 3 orders of magnitude. In fact, the resistance calculated in this work is much higher than that which is calculated for the single graphene sheet, Rs ∼ 275 Ω per square, or for four-layer, ∼ 40 Ω per square, that fabricated from the CVD method [50,51]. These results illustrate that, the non-uniformity of the graphene layers and the low conductive polymer-graphene composites significantly increases the sheet resistance in graphene films. In general, increasing the surface area and crystal quality of graphene sheets reduces the Rs of graphene films. Nevertheless, the sheets resistance of the graphene films prepared by these methods was comparable to other electrical measurements of polymer-graphene composites [52].

**Table 1:** Sheet resistance results of SP thin film with and without graphene.

Material	Rs (Ω per square)
**SP**	<10^6^
**SP-G**	1.5×10^3^

### Thermal diffusivity enhancement of graphene solution

**Fig 10:**
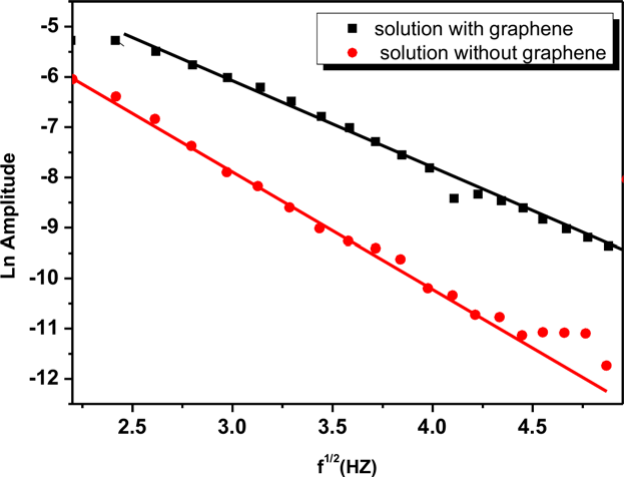
Variation Ln amplitude of PE signal as function of the square root of frequency for SP solution with and without graphene.

The enhancement in thermal diffusivity of the SP-G solution was analysed, by first determining the thermal diffusivity of DI water as a reference liquid. The plots in S2 Fig show the signal Ln amplitude and phase as a function of the square root of the frequency for DI water using Eqs (1) and (2). The calculated average value of thermal diffusivity of water from PE amplitude and phase was 1.444±0.030×10^−3^ cm^2^/s, which differed from literature values by less than 2% [39]. The thermal diffusivity measurements of the SP solution with and without graphene are summarized in Fig 10 and Table 2. This result showed that the thermal diffusivity of the solution with graphene, SP-G, was enhanced by a factor of 3.

**Table 2:** Summarized results for thermal diffusivity of SP solution with and without graphene.

Material	Thermal diffusivity (cm^2^/s)×10^−3^
**SP**	0.813 ± 0.110
**SP-G**	2.544 ± 0.056

To the best of our knowledge, there are no reports on thermal diffusivity of the exfoliated graphene nanofluids. Therefore, this method to measure thermal diffusivity of the graphene nanofluids may provide a new Insight to measure different properties of graphene nanofluids. This enhancement is in agreement with other reports in the literature for thermal conductivity of the graphene composites [53]. While, the thermal diffusivity of graphene nanofluid is not available in the literature, some good amount works have been carried out in the literatures to investigate thermal conductivity of graphene solutions in a variety of solvents [46, 54–58]. Accordingly, the thermal conductivity of the suspension is represented by *K*_*n*_ = *α*_*n*_(*ρ*_*n*_*C*_*n*_) Where *α*_*n*_, *ρ*_*n*_ and *C*_*n*_ are the thermal diffusivity, density and specific heat capacity of the nanofluids. It can be seen that in low concentration of grapheme obvious enhancement in thermal diffusivity can be expected with similar enhancements in thermal conductivity. Table 3 shows the comparison between carbon-based nanofluids in some of the recent works. From this, it is evident that the enhancement in thermal properties of graphene nanofluids (GN) is found to be very significant at the surface area of the graphene exfoliated in the solution due to well dispersion of graphene sheets and high stability of graphene nanofluids.

**Table 3:** The comparison between thermal conductivity enhancements of carbon-based nanofluids in some of the recent works.

Particle type	Base fluid	Concentration (wt%)	Enhancement (%)	Reference
**GN**	DW	0.056	64	[54]
**GN**	DW	0.1	27.6	[55]
**GO**	EG	12	61	[56]
**MWNTs**	DW	0.6	34	[57]
**Graphite**	DW+PVP	0.5	23	[58]

These results confirm that the solution containing graphene had a large scale exfoliation into single or few layer of graphene that can be good candidates for high thermal conduction in graphene or graphene composite devices. The physical and chemical properties of graphene depend on its crystalline structure along with the number of layers. The combination of TEM observations along with, UV-vis absorption, conductivity measurements, and thermal properties clearly indicate a dispersed graphene solution with high stability, using this environmental solvent. The lack of any individual single layer graphene sheets suggested that the graphite cannot be fully exfoliated. Separation of graphene layers depends on the mechanical disruption of the graphene stacking by ultrasonication [17,18]. In order to achieve single or double layered graphene, graphite needs to have a fully exfoliated structure. The images in Fig 4 shows that ultrasonication treatment has a significant influence on the exfolation of SP-G solution. Consequently, further increase in the ultrasonication time, leads to improve mechanical exfoliation of graphene sheets [40]. In this case, the strong interaction between graphite flakes with polymer molecules of soap/PVP can reduce the electrostatic effects and prevent graphene aggregations. A good dispersion of graphene was obtained in water by using soap as surfactant with PVP as a stabilizer followed by ultra-sonication. Thus, this is a highly desirable method due to the low cost of the surfactants which yields an excellent production of exfoliated single and layered graphene. This significantly high and well-dispersed graphene sheets were then used to improve the photonic, mechanical, thermal and electrical properties of graphitic materials.

## Conclusions

In conclusion, a simple liquid exfoliation method of producing layered graphene with a low defect ratio from pure graphite powder was obtained by a sonication treatment in a mixture of SP surfactant in DI water. The presence of SP solution in this technique allowed an excellent dispersion of graphene sheets with a high stability, even after 6 months. In the thermal diffusivity of the SP solution with exfoliated graphene demonstrated a precise enhancement (by a factor of 3). This enhancement compared favourably to other graphene-loaded thermal conductive graphene composites. The fabricated film from SP-G solution containing graphene was utilized to make an electrical conductive polymer-graphene film composite, the sheet resistance was found to be 3 order magnitude lower than that of SP solution. Graphene with a precise dispersion was obtained from this technique, without any oxidization or significant defect formations. The grapheme produced is very promising for the development of the graphene based biomedical and electrical applications.

## Supporting Information

S1 Fig Schematic diagram of TWC technique. More information about TWC technique
(DOCX)

S2 Fig **The frequency behavior of the Ln amplitude and phase of PE signal obtained from DI water** (a) ln (amplitude), and (b) phase of PE signal versus the square root of the frequency for one of DI water.
(DOCX)
