# Supplementary material for: Environmental Synthesis of Few Layers Graphene Sheets Using Ultrasonic Exfoliation with Enhanced Electrical and Thermal Properties
Source: PLoS One. 2016 Apr 11;11(4):e0152699. doi: 10.1371/journal.pone.0152699 (PMC4827812; doi:10.1371/journal.pone.0152699)
Supplement: S1 Fig — More information about TWC technique (DOCX) [file pone.0152699.s001.docx]

The TWC technique mostly has been applied to measure thermal diffusivity of liquid sample. In this technique, the liquid sample and the PVDF detector should be in a thermally thick regime, in order to have the information of sample near its surface [33]. At low frequency the sample is in thermally thin regime, with increasing the frequency the effect of thermally thick becomes obvious, at high frequency the anomalous signal becomes very small and rather frequency independent. So, in this work, the useful frequency range was between 5-25 HZ, the experimental measurement was performed at room temperature (~22°C). The operating parameters were controlled through a computer equipped with a LabVIEW software, to capture the amplitude and phase data and the data were analyzed using Origin 8. A schematic of the experimental apparatus is shown in S1Fig.


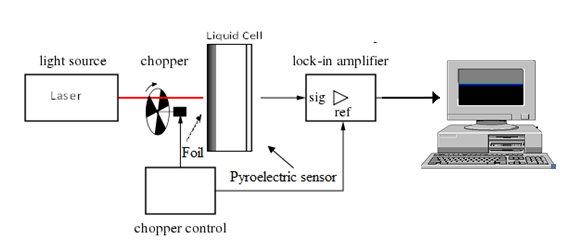


**S1 Fig.**
